# Ten simple rules for empowering women in STEM

**DOI:** 10.1371/journal.pcbi.1010731

**Published:** 2022-12-22

**Authors:** Patricia Guevara-Ramírez, Viviana A. Ruiz-Pozo, Santiago Cadena-Ullauri, Gabriela Salazar-Navas, Ana Acosta Bedón, J. Faustino V-Vázquez, Ana Karina Zambrano

**Affiliations:** 1 Centro de Investigación Genética y Genómica. Facultad de Ciencias de la Salud Eugenio Espejo, Universidad UTE, Quito, Ecuador; 2 Department of Cell and System Biology, University of Toronto, Canada; 3 Escuela Nacional de Ciencias Biológicas, Instituto Politécnico Nacional, Ciudad de México, México; Dassault Systemes BIOVIA, UNITED STATES

## Introduction

Gender differences have been around for many decades. In consequence, women demanded equal rights, achieving changes in education and publications of their works through revolutionary and social movements [1]. However, there is still evidence of gaps, reflected in women’s underrepresentation in jobs, institutions, and education, so that gender inequality persists [2].

For example, according to the progress on the sustainable development goals (SDGs), with a gender snapshot worldwide for 2021, women hold just 28% of jobs in science, technology, engineering, and mathematics (STEM) [3].

**Fig 1:**
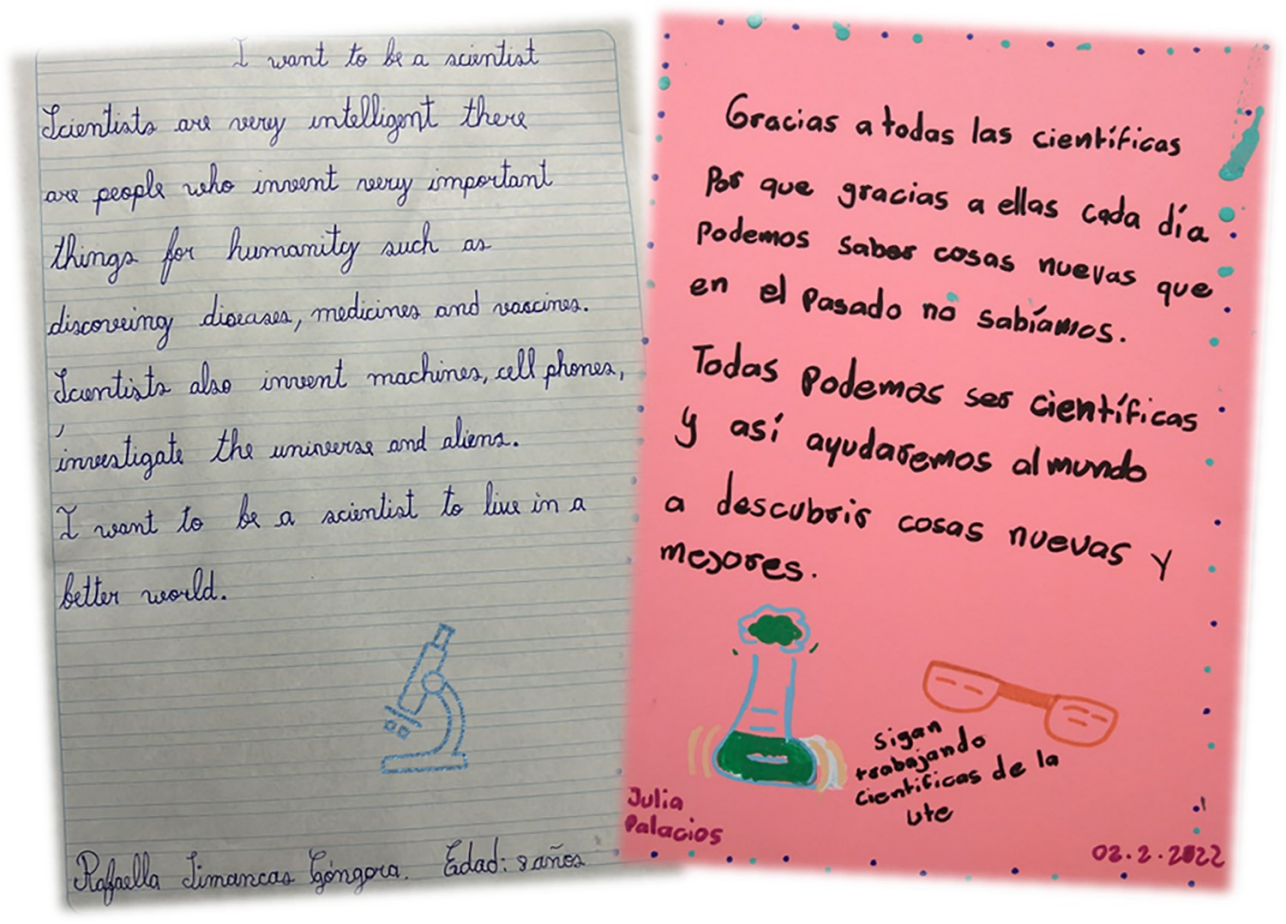
Letters of girls interested in science (English and Spanish) (Written by Rafaella Simancas and Julia Palacios).

**Fig 2:**
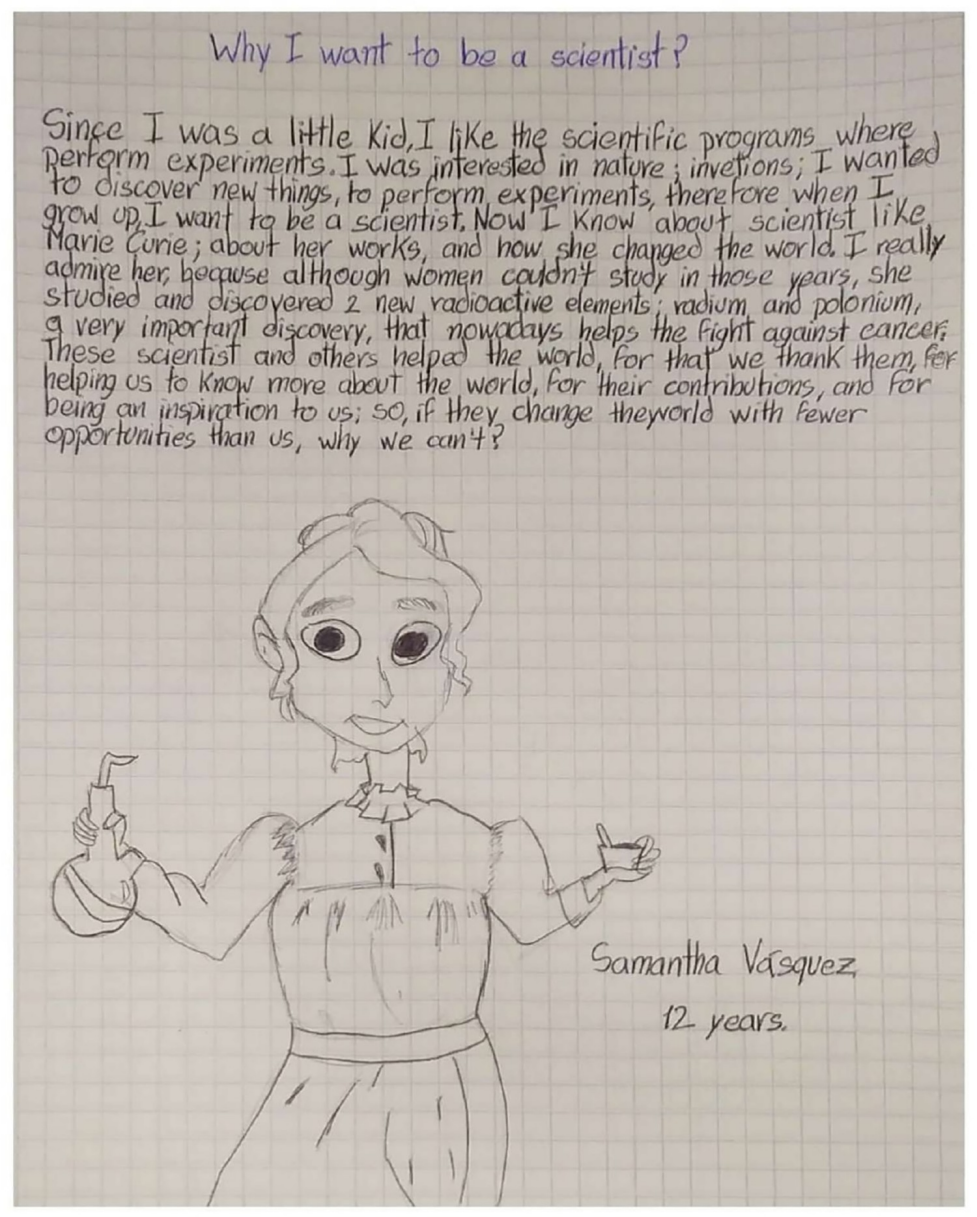
Letters of girls interested in science (Written by Samantha Vásquez).

Despite these obstacles, the research work from women in STEM has shaped the scientific understanding in many fields and continues to do so with present-day contributions such as the Coronavirus Disease 2019 (COVID-19) vaccine designed by Sarah Gilbert [4] or the genome editing technique by Jennifer Doudna and Emmanuelle Charpentier [5]. Likewise, achievements in geometric partial differential equations by Karen Keskulla Uhlenbeck, who was the first woman to win the Abel Prize for Mathematics in 2019 [6], or the discovery of a supermassive compact object at the galactic center by Andrea Chez can be mentioned [7]. In addition to these recognized women, many more continue to work for the benefit of science, inspiring girls worldwide. For example, Ecuadorian girls have sent letters to scientific women expressing their love for science (Figs 1 and 2).

Despite these significant contributions, women still receive less recognition and awards. A total of 616 Nobel Prizes were awarded between 1901 and 2019 in Physics, Science, Medicine, and Physiology; however, only 19 were awarded to women.

While some changes have been accomplished to reduce women’s underrepresentation in STEM, gender bias still exists nowadays. Consequently, this article poses ten simple rules on how society could change and prevent gender differences to empower women in STEM.

## Rule 1: Avoid the “Matilda Effect”

In 1993, the science historian Margaret Rossiter coined her most famous expression, the “Matilda Effect,” to describe the bias that denies recognition to women’s work. This term is in honor of Matilda Joslyn Gage, the first activist who fought for women’s rights and published a landmark article titled “The Matthew Matilda Effect on Science” [8].

While women have gained significant ground in the scientific world, there is still a lack of recognition. For example, enterprises tend to hire men, rate them better in their applications, and pay them higher salaries [9]. Furthermore, reviewers tend to give positive comments on scientific papers in which men are the first author [10]. There is also a disproportionate number of citations to studies written by female scientists and fewer award nominations and collaboration opportunities for women [11]. Thus, it is important to eliminate the “Matilda Effect” to ensure the recognition of women’s work and scientific achievements.

One simple initiative could target mentors to encourage female researchers to seek awards and come up with candidates for recognition or leadership positions. Similarly, before selecting the candidates, the selection criteria should be gender-blind. Anonymizing applications could ensure that the ratings reflect the merits of the postulants and mitigate gender bias in the review [12–15].

Moreover, for scientific publications, the academic leaders could determine authorship based on the contributions, and editors of scientific journals should analyze data regarding the gender breakdown of papers and promote the awareness of the editorial staff to work on initiatives to overcome gender bias.

**Fig 3:**
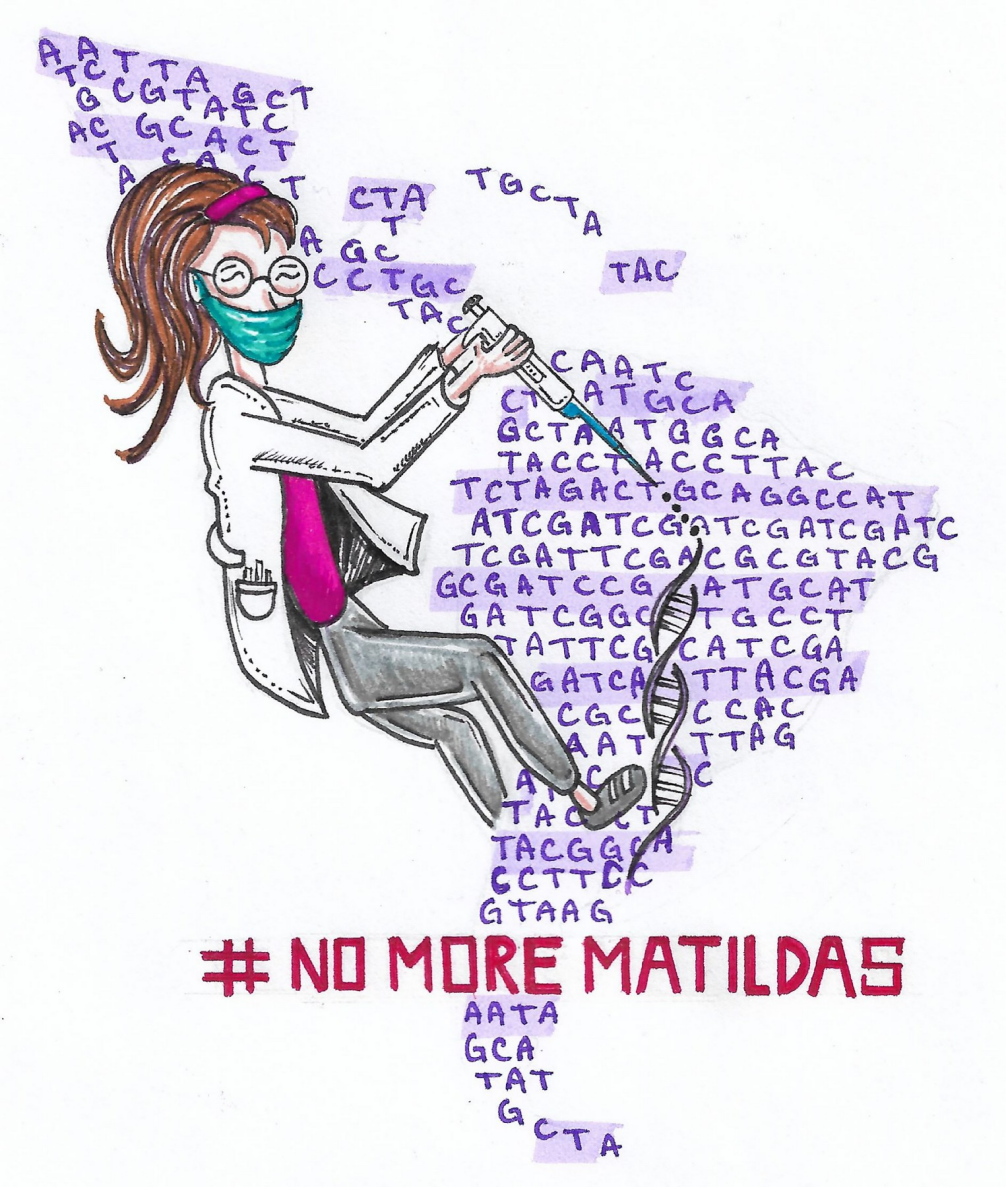
Avoid the “Matilda Effect”. To increase the recognition of women researchers, the “Matilda Effect” must be avoided (Illustrated by Jose Faustino Villa).

Finally, other solutions to mitigate this effect include: providing gender inclusion workshops in organizations to help build an inclusive workplace, lead to more gender-neutral hiring, and actively share their scientific work in different media such as social networks, blogs, conferences, and others to let society know what they are doing for science worldwide. To sum up, to abolish the “Matilda Effect,” governments and institutions should work on an inclusive society with initiatives to ensure the acknowledgment of women’s contribution (Fig 3).

## Rule 2: Empower other women through solidarity between them

On several occasions, women have faced discrimination, racism, inferiority, and even competition or exclusion among women themselves [16,17]. Throughout history, according to Amelia Valcárcel, some relationships between women have been quite complex, creating difficulties such as power differentials, hierarchies, supremacism, sabotage, competition, and rivalry [18].

**Fig 4:**
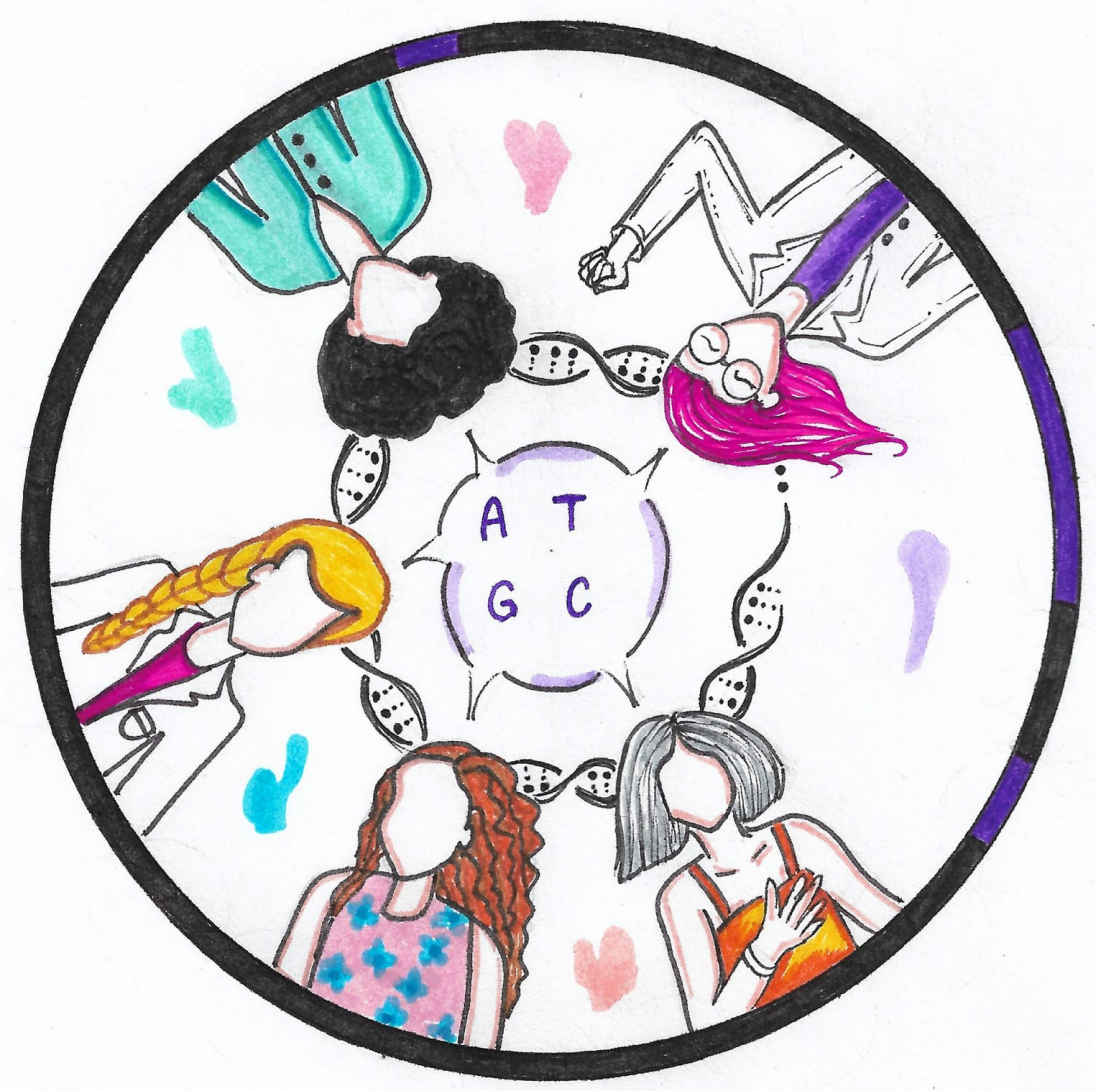
Empower other women through solidarity between them. A group of women joined together by common interests (Illustrated by Jose Faustino Villa).

Solidarity between women is bound by shared ideals to build trust, reciprocal recognition, and support for one another, enhanced by women’s teams cooperating throughout the various stages of the research career (Fig 4). These groups of women allow sharing of professional knowledge and experiences to develop skills effectively [19]. For example, in January 2018, the platform Request a Woman in STEMM (science, technology, engineering, mathematics, and medicine) was created by a cohort of 500 women scientists. This platform connects a broad multidisciplinary group of women in science, allowing the community to contact them [20].

Other initiatives are the program “Women and Engineering” of the Royal Academy of Engineering, which promotes STEM vocations based on the experience of women working in these areas [21], and the “Girls in Tech” organization helps to connect and learn from other women in technology via mentorship programs, community events, and online discussions. Likewise, “CODE2040” creates opportunities for underrepresented women minorities in technology [22].

By establishing solidarity between women as an empowerment strategy, great motivation can be obtained. It is essential to work as a team and share knowledge to grow together, instead of disregarding each other and considering each other as rivals [19,23,24]. Thus, to empower women through solidarity, bridges of dialogue must be built, creating interpersonal relationships with other experienced women who can give guidance and advice.

## Rule 3: Collaborate with other research groups, organizations, and institutions

In STEM careers, collaboration between researchers is essential to address perplexing questions and solve complex problems that arise in research. Cooperation within the work team and with other groups is indispensable to developing research with an interdisciplinary and multidisciplinary approach [25]. Research must be open to interacting with different sciences and carrying out studies at an international level for the mutual development of underrepresented regions like Latin America.

However, studies mention that differences in collaboration based on gender bias can affect the effectiveness of team building, recruitment, and retention of researchers [26,27].

Helicopter research is another problem. This is a practice in which scientists from wealthy countries take samples, and materials from lower-income countries, publishing the results with little involvement from the local (women) scientists. Women may be more vulnerable to these types of science because of their desire for collaboration [28]. Consequently, unequal collaborative research practices are based on gender or resource appropriation.

Solutions to abolish these problems are: (a) promote collaborative research practices from the beginning and not perpetuate colonization practices [29]; (b) establish an agreement that outlines equal scientific partnership [30]; and (c) consider the women who have been part of the research when establishing the collaboration.

**Fig 5:**
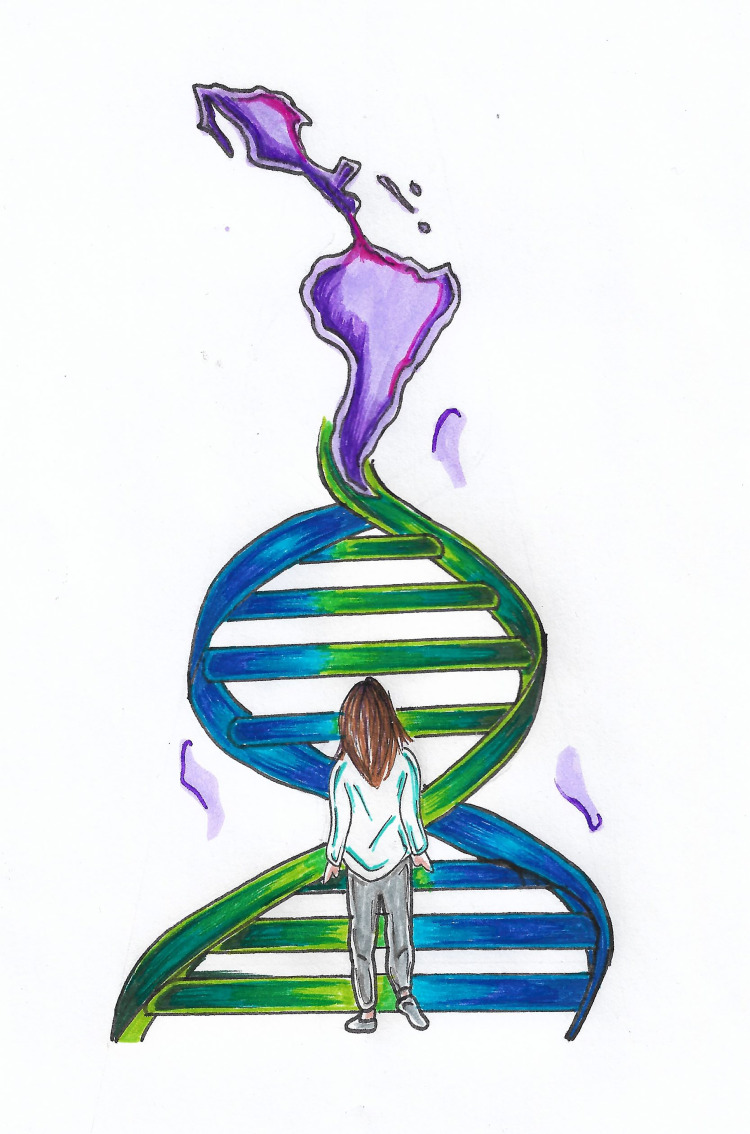
Collaborate with other research groups, organizations, and institutions. Knit educational programs, research, networks, and other international initiatives designed to promote fair and equitable collaboration (Illustrated by Jose Faustino Villa).

Finally, recent studies suggest that collaboration could be improved by the presence of women in the research team. Hence, promoting women’s participation in STEM staff could increase scientific productivity and the quality of the collaborations (Fig 5).

## Rule 4: Research and publish

**Fig 6:**
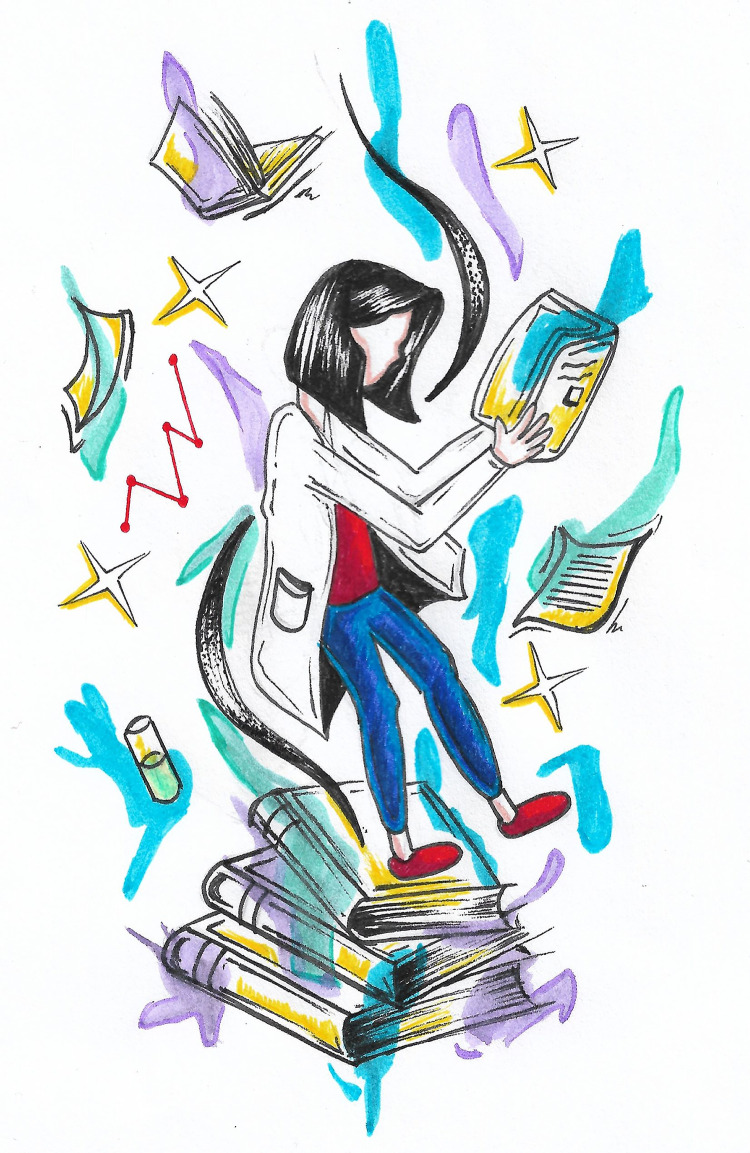
Research and publish. Women scientists require more actions that promote the publishing of their research (Illustrated by Jose Faustino Villa).

Scientific publications are essential to demonstrate professional growth in the field (Fig 6). Since 1994, there has been an increase in women as the first authors of publications. However, the COVID-19 pandemic showed a decrease in women’s scientific contributions [31].

In the STEM profession, publications and grants are the most crucial metrics for recognizing the impact of science [32]. There are studies on gender bias in research and publication, which describe: (a) men are invited for paper submissions at double the rate of women [33]; (b) male scientists have an average of 3.6 more papers than female authors, consisting of a 27% gap in productivity [34]; (c) articles published by male scientists receive 30% more citations than female scientists [34,35]; (d) a negative correlation between journal impact factor and women representation [36,37]; and (e) a fewer percentage of females than males in journal editorial boards [38]. Thus, women in STEM need to be encouraged and supported throughout the publication system.

Actions like increasing the funding for grants and publications for women and double-blind reviews can be strategies to increase women’s authorship representation. Another way to promote women’s publications is by organizing workshops and congresses for female-led research [39,40].

Similarly, the Declaration on Research Assessment (DORA) aims to raise awareness in the use of metrics that align with core academic values, develop new policies for decision-making, and spread research [41–43]. Adopting DORA promotes the reduction of the existing gender gap and equity. DORA should be implemented in institutions to empower better career metrics for women researchers.

## Rule 5: Ask for help

**Fig 7:**
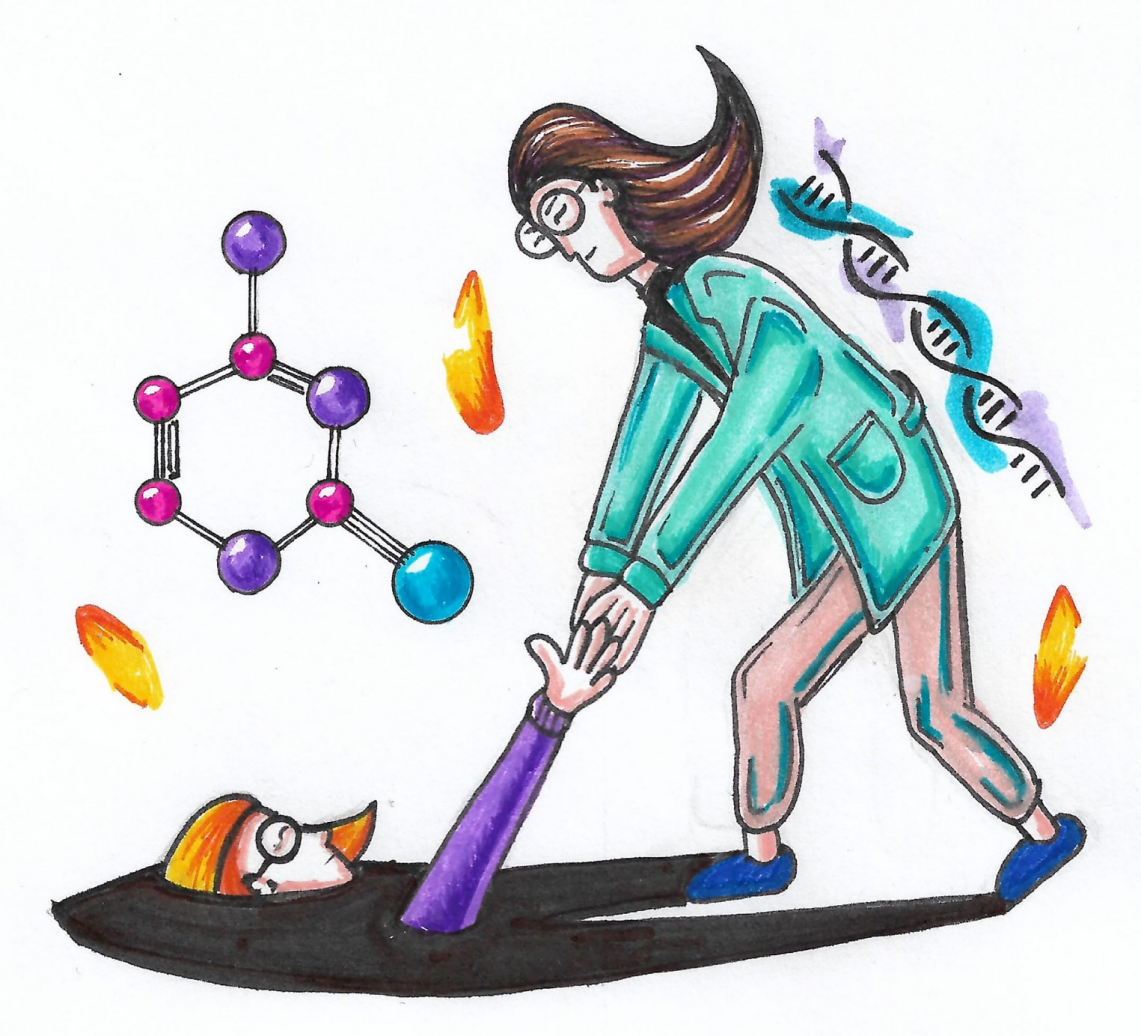
Ask for help. Researchers help researchers to develop science and be part of the scientific world (Illustrated by Jose Faustino Villa).

Starting a new job or developing a new project with a close deadline could make women feel anxious, but there is one clear solution: ASK FOR HELP (Fig 7).

Being an independent women scientist does not mean doing everything on your own; there could be stages of a process that a scientist does not fully dominate. When there are doubts, it is important to work together and ask for help from people who have more expertise in the area [44].

A research group may be a particularly uncomfortable space, where asking for help is challenging, since it is a place where the researchers are trying to demonstrate as much experience, competence, and confidence as possible. However, asking for support could mean improved performance and continued professional development gained from advice, referrals, and resources [44,45].

Some estimates suggest that up to 90% of the help received from colleagues is by direct request. Therefore, it is necessary to formulate the request effectively, concisely, and clearly. Furthermore, it is important to use the correct language and to acknowledge the benefits that the help could have on the final product [45].

Women asking for help will become more resilient and successful researchers.

## Rule 6: Stop the prejudices—Do not feel different for being a woman

Women may feel inferior just because of being a woman. The Women in Science and Technology Equal Opportunity Act declares that men and women have equal opportunity in education, training, and employment in scientific and technical fields [46]. However, evidence suggests that people may perceive scientists as more like men than women. In this context, more pictures of male scientists are seen in science magazines. Furthermore, in experiments using the Draw-a-Scientist test, in which children of different ages participated, they drew a higher percentage of men as scientists [47,48].

This problem is further exacerbated by some unconscious behaviors of parents and teachers who promote the thinking that certain STEM disciplines are more suitable for men. The effect of implicit stereotyping of children from a very early age can differentially influence interest, achievement, and persistence in particular sciences, such as the field of genetics, molecular biology, engineering, and other sciences involved [49].

In addition, there are negative stereotypical perceptions of women based on their appearance, clothing, how much makeup they have, and other banal aspects not related to their work in science [47].

**Fig 8:**
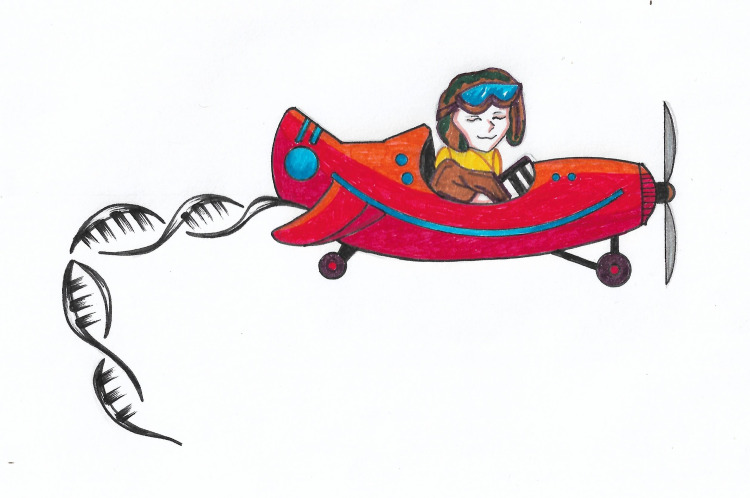
Stop the prejudices. “The most difficult thing is the decision to act. The rest is merely tenacity”—Amelia Earhart (Illustrated by Jose Faustino Villa).

One solution is to create support centers for women and open spaces to discuss these issues. Additionally, gender equality training should be introduced at all levels of education since it would help to reduce gender bias, harassment, and the persistence of negative stereotypes. These education programs are essential to show children that men and women can perform notably in STEM fields and do science [47]. But the most important part of the change starts with women, who should believe in themselves as one way to demonstrate that males and females are equal and stop prejudices against women (Fig 8).

## Rule 7: Encourage STEM education

One of the most challenging decisions in a person’s life is career choice. Many teenagers are often confused and tend to fall into stereotypes, believing that professions depend on gender, social identities, and self-concepts. Even though there are STEM women role models, who could impact positively on girls’ career decisions, it is crucial to start empowering them at an early age [50,51].

Gender differences in STEM participation are visible from childhood and continue throughout academic life. For example, women represented only 35% of students enrolled in STEM disciplines in higher education in 2017 [52]. Women’s underrepresentation in STEM education could result from the interaction of social, cultural, psychological, and learning process barriers [50].

Moreover, studies suggested that family and teachers tend to encourage more mathematics, science, and technology learning in boys than in girls through play opportunities, games, or toys during childhood [53]. Research in toy-buying patterns revealed that it is common to give science-related toys to a boy, a stereotype that might incline boys toward science careers [54]. Thus, the aforementioned factors could influence women’s career choices, depriving them of getting access to STEM studies.

The interventions to solve the gender education–STEM gap include building interest and motivation in girls by developing science skills in early life and expanding family understanding of STEM education opportunities connecting girls with quality education. At the school level, the efforts must impact curricula to include gender gap studies in the society. Furthermore, countries should focus on making social and cultural changes in their legislation, allowing and motivating women to access STEM education [28,55].

**Fig 9:**
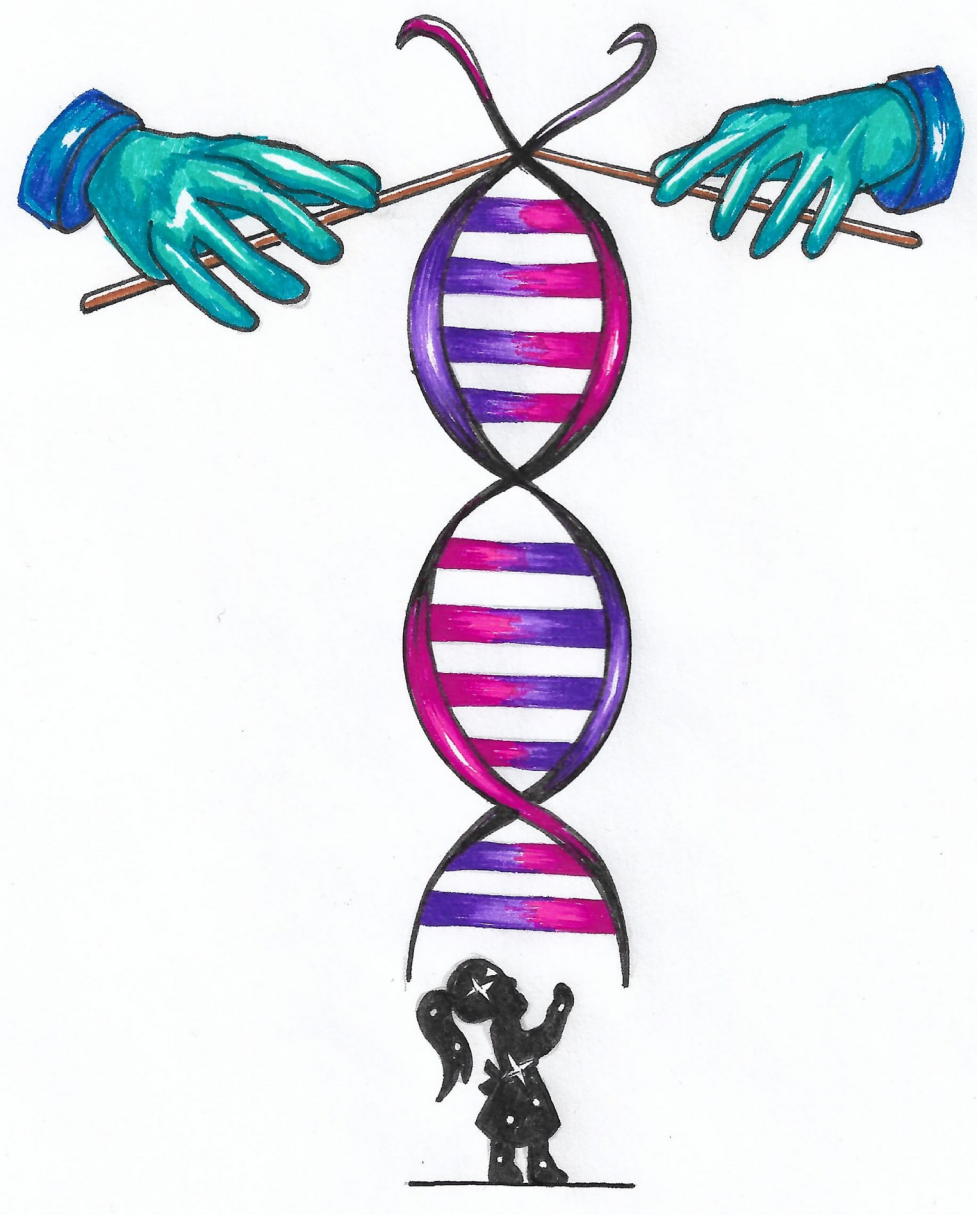
Encourage STEM education. Girl building her education in STEM (Illustrated by Jose Faustino Villa).

Therefore, women must be empowered and guided from the beginning of their educational life (Fig 9). Since, studying science in any of its specialties is a life decision that requires quality education, different governments and foundations should offer scholarships for academic training.

## Rule 8: Balance time to perform roles in society and family

Modern women have several roles in society and family, and combining them with professional STEM careers can be challenging [56].

There are gender biases that impact women’s careers in the balance of work and family. Parenting kids directly affects women in science; not even the resourceful countries offer the most basic support for women with children. Consequently, caregiving typically becomes the mother’s responsibility, limiting the research and study time [57,58]. Every role is important for society, so the government and academia should implement effective policies that allow progress in the SDGs with the gender snapshot [58].

Studies have reported recommendations to achieve a better work–life synergy. Some examples are: (a) introducing child and elder care facilities; (b) work time analysis to reduce the overload with the possibility of hiring additional personnel; (c) strong colleague relationships in the company for effective teamwork; and (d) planning training on time management in the annual capacitation plan; among others [58].

In addition, personal endeavors to balance time are: (a) establishing priorities among tasks and roles; (b) planning and scheduling work, personal, social, and familial activities; (c) building boundaries between work and social activities; and (d) organizing vacations; among others [59–63].

**Fig 10:**
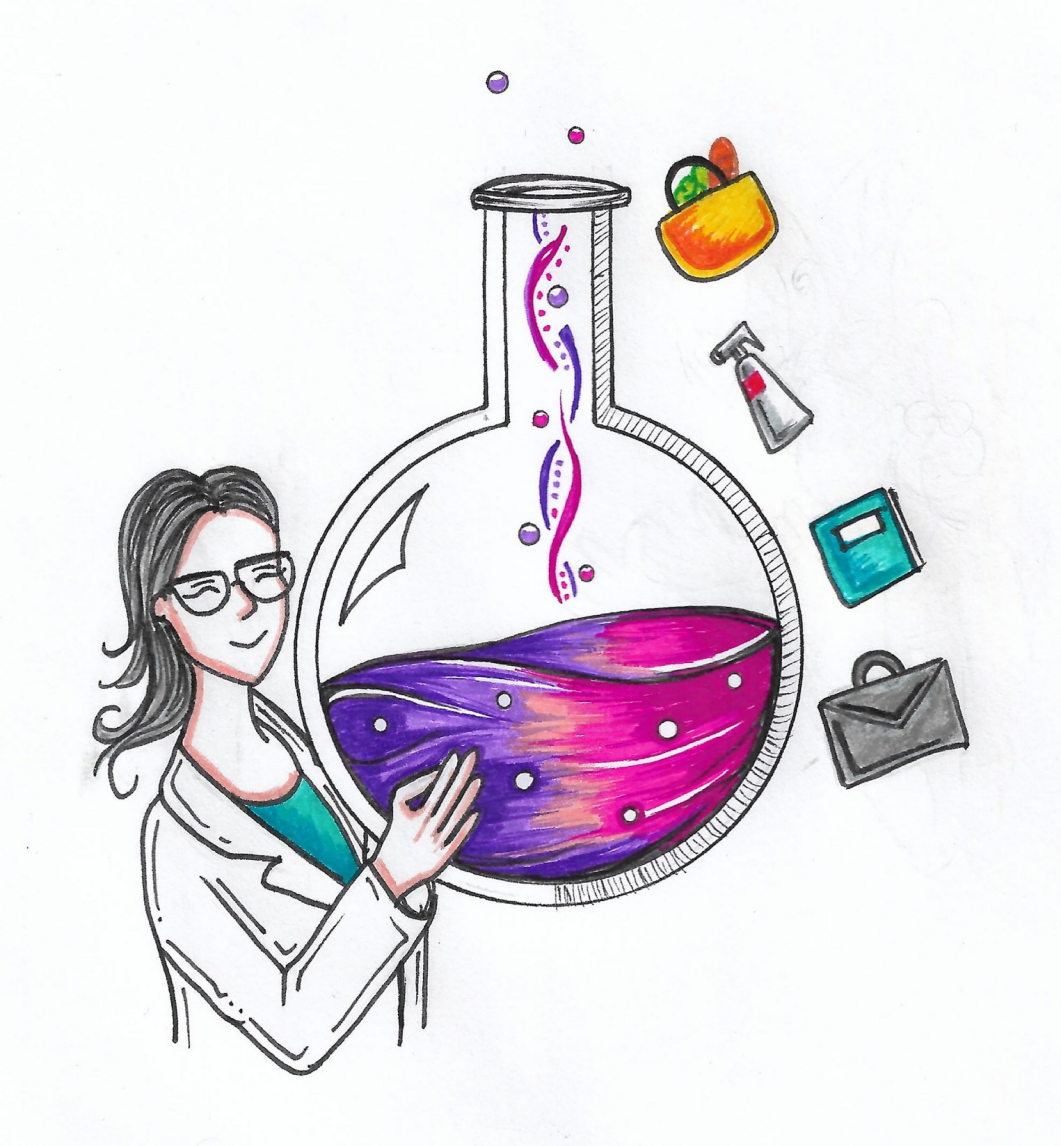
Balance time to perform roles in society and family. Women scientists balance their time to perform the different roles in society and family (Illustrated by Jose Faustino Villa).

The work–life balance constitutes an indicator of life satisfaction. The key is to learn how to manage time-limiting job activities and leave enough time for themselves and their social and family lives (Fig 10).

## Rule 9: Remember that it is never too late

Internal or external factors might lead our career pathways to unexpected places we did not plan. The rule “it is never too late” highlights the diversity and limitations that have led women to start their careers and motivates them to request initiatives that focus on their professional growth.

Life experiences could persuade women to stop following their dreams in STEM, like (a) early marriage or motherhood, where money prioritization does not allow them to study [64]; (b) some religions’ cultural norms and doctrines affect a woman’s educational career choices [65]; (c) unpleasant experiences with teachers of science-related subjects could demotivate them [66]; (d) choose a career other than one in STEM thinking that is more suitable to their interests [67]; and (e) scholarship and study positions limited by age. These reasons are why some women and young girls might not apply for a STEM program, even if they are attracted to it.

To solve these issues, provide information on STEM careers, aptitude tests, and psychological counseling to women to analyze their career goals.

**Fig 11:**
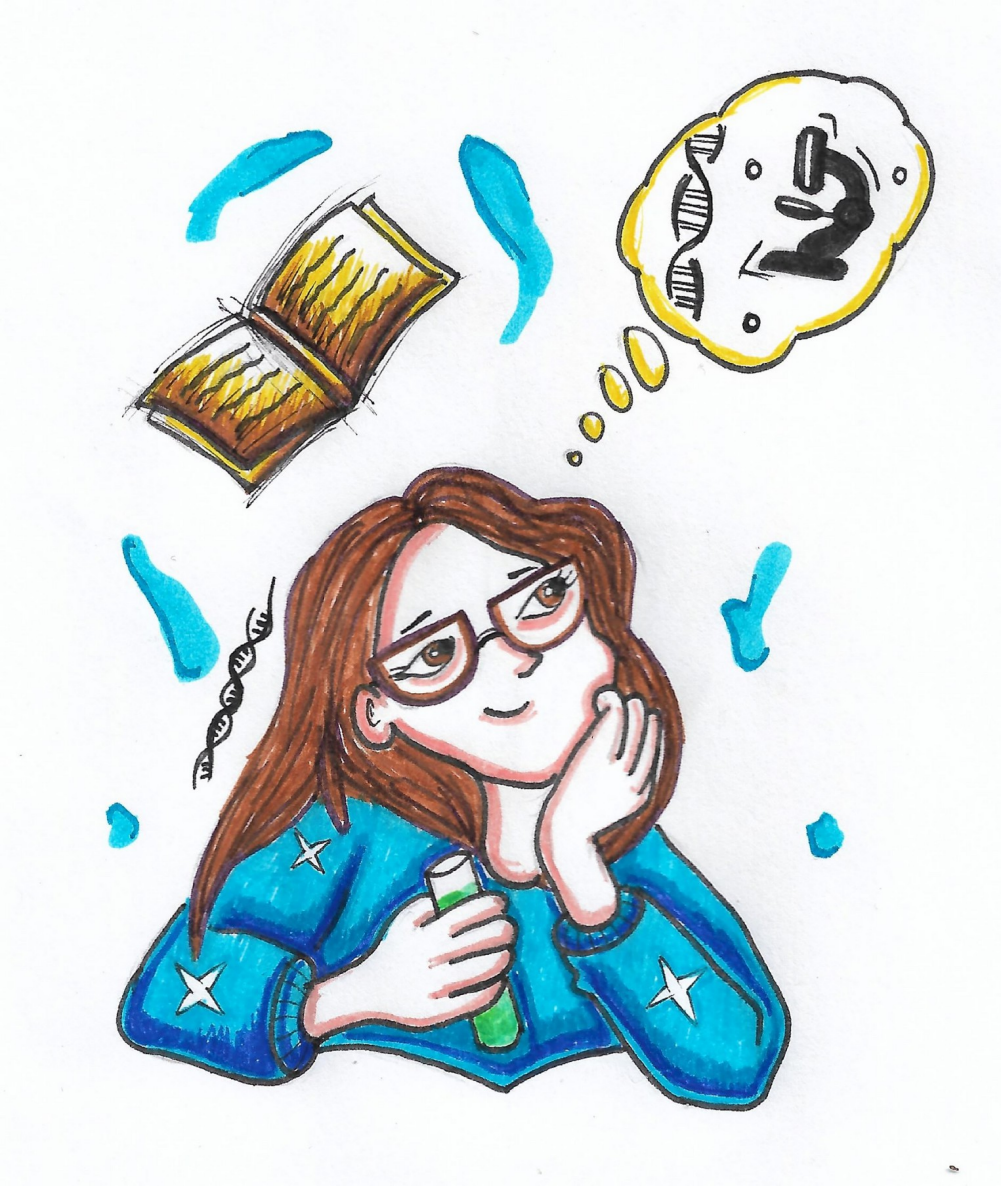
Remember that it is never too late. Every step counts while we are building a way to reach our purposes (Illustrated by Jose Faustino Villa).

Moreover, it is important to reconsider the academia application requirements without limiting scholarships or positions to certain ages. It is never too late to change or create pathways for a fulfilled professional life (Fig 11). Hence, it is crucial to take some time to recapitulate and decide if you are in the place you want or at least if your decisions are leading you there.

## Rule 10: Remember our capacities and strength

**Fig 12:**
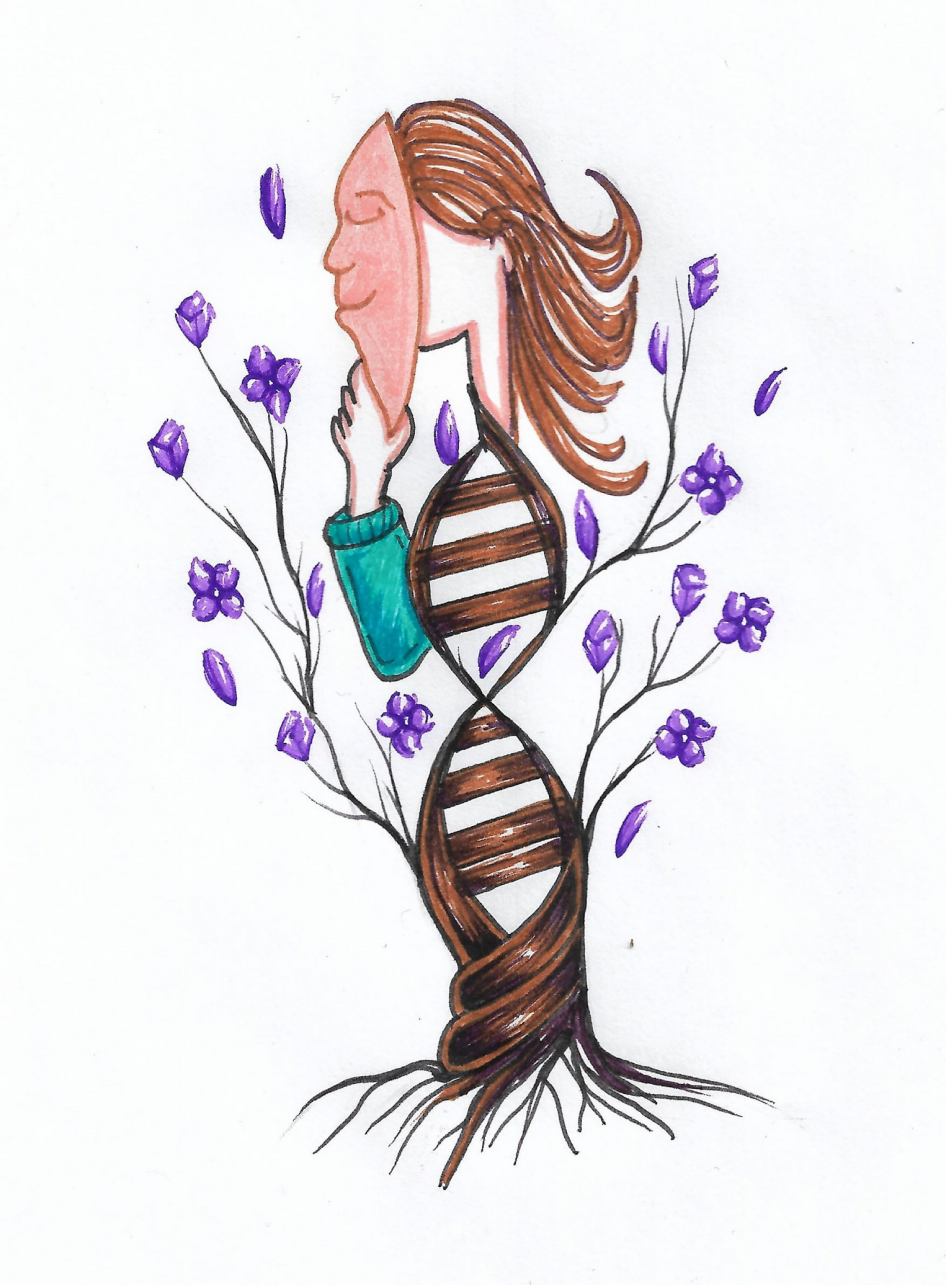
Remember our capacities and strength. Do not mask your capacities; remember that all your success is because of them (Illustrated by Jose Faustino Villa).

STEM professionals who believe brilliance is required for success are more likely to doubt their capacities. Doubting one’s abilities and constant fear of being exposed as a fraud is a pattern of thinking known in psychology as the Impostor Syndrome. This psychological phenomenon does not depend on external evidence of success but rather on a state of mind in which a person believes it is not enough [68] (Fig 12). The characteristic effects of this syndrome are: attributing the success to external factors; fear and guilt about success or not living up to expectations; self-doubt or denial of your competence and skills; refusing praise, and sabotaging your success [69].

In STEM, imposter syndrome is common because of the rapid technological advancement and change, which no one could ever keep up with, but sometimes researchers feel they should [70]. Studies estimated that 70% of people have suffered at least one experience of the impostor phenomenon affecting both men and women [71,72]. Nevertheless, a study on 5,000 STEM students and graduates found that women are more likely to feel they do not deserve recognition for their achievements [73].

Methods to overcome the impostor syndrome are focused mainly on recognizing your potential since women have always been resilient and capable of doing their job [74]. Some suggestions are: celebrate your achievements; utilize additional support (friends, therapist); remember that perfectionism is subjective [75]; stop comparing with others; identify when it occurs; set measurable goals; and, lastly, positive thinking.

Mental and physical health are necessary to develop capacities and strength; therefore, women must change their internal talk and external influences to motivate and remind themselves about what they can achieve.

## Conclusions

These rules may not be the only ones for being an empowered STEM women researcher. However, we hope our ideas and experiences will help readers to realize the need to build an equitable STEM community.

Our expectation with this article is that it will serve as a basis for governments, institutions, and academia to generate policies that contribute to the participation of women in science. Such policies could open the range of possibilities for women from an early age to decide their professional future with freedom, without fear of failure, and without considering unconscious bias.

Science and technology should no longer be deprived of the potential that women have.
